# Virtual & mixed reality fatigue questionnaire

**DOI:** 10.1186/s12909-026-08641-w

**Published:** 2026-02-01

**Authors:** Ana María Cintora-Sanz, Raúl Muñoz-Romo, Helmut Schrom-Feiertag, Alberto Blanco-Lara, Tatiana Vazquéz-Rodriguez, M. Carmen Cardós-Alonso

**Affiliations:** 1 Prehospital Medical Emergency Service of Madrid Community SUMMA112, C/Antracita, nº2 bis, Madrid, 28045 Spain; 2 Department of Artificial Intelligence and Human Interfaces, Austrian Institute of Technology Center for Technology Experience AIT, Giefinggasse 4, Vienna, 1210 Austria

**Keywords:** Virtual reality, Fatigue, Questionnaire, Training, Emergencies training

## Abstract

**Background:**

Virtual Reality (VR) generates an artificial environment in which users interact with computer-generated scenarios and sounds. Using devices such as headsets and motion sensors, users are immersed in a simulated world. Augmented reality (AR) is an interactive overlay of the real environment that provides an additional wrapper over the environment, and the user experiences an immersive and interactive environment. In Mixed reality (MR), VR and AR elements are combined; computer graphics interplay with real objects, allowing users to interact with virtual and physical objects at the same time.

VR and MR are effective training tools in different healthcare settings. These tools are useful for preparing emergency health personnel to respond to disasters by providing them with an immersive reality since real practices are very difficult to implement in mass casualty incidents.

Given the usefulness of this approach, assessing the optimal training times associated with this method and the side effects that may influence learning is helpful. One of the most common side effects is fatigue. We developed a questionnaire to assess fatigue in terms of various dimensions, that can affect a person while training in the context of VR and MR.

**Methods:**

We designed a questionnaire to assess the fatigue levels perceived by professionals and validated this measure after VR training experience. We analyzed ratings of visual, mental, physical, and general fatigue. This questionnaire was applied to emergency professionals (Sample size = 101).

**Results:**

The reliability and validity of the questionnaire were assessed in terms of the following factors: general, social, emotional, visual, and motivational fatigue.

**Conclusions:**

The results of this research suggest that fatigue is an element of VR and MR training. Educators should take these effects into account to optimize learning in the context of VR and MR. According to user feedback, the optimal length of time for mixed reality training is around 20 min, especially when the user has little or no experience.

**Supplementary Information:**

The online version contains supplementary material available at 10.1186/s12909-026-08641-w.

## Highlights

- Headset-based virtual training simulation modalities yield fatigue symptoms.
- The Virtual and mixed reality fatigue questionnaire can value the different fatigue profiles.
- Virtual reality training can cause visual fatigue, general fatigue, and decreased concentration afterwards.
- The optimal time for mixed reality training is about 20 minutes, especially when the user has little prior experience.

**Supplementary Information:**

The online version contains supplementary material available at 10.1186/s12909-026-08641-w.

## Introduction

### Background

It is becoming increasingly evident through different studies that extended reality (XR) technologies can have unintended consequences [1]. In terms of defining the scope of this transformative tool in the digital landscape, it is needed to explore the definitions of the included fields under the spectrum of extended reality (XR).

Augmented Reality (AR) is a technology that overlays digital information onto the real world, significantly enhancing user interaction and experience. It has evolved from its early beginnings in the 1960 s, to the modern era where mobile devices and advanced sensors enable immersive applications across various fields, including education, healthcare, and retail [52]. In contrast, Virtual Reality (VR) immerses users in entirely digital environments, while Mixed Reality (MR) blends elements of both AR and VR, allowing for real-time interaction between physical and digital objects. This differentiation underscores the unique capabilities and applications of each technology, establishing AR, VR, and MR as new technologies with notable impact across diverse sectors:

Virtual reality (VR) has become increasingly prominent as an effective training tool in different healthcare settings [6, 23, 33] since it was first introduced more than 56 years ago [18].

Head-mounted displays (HMDs) enable the application of virtual reality (VR) and mixed reality (MR) technologies in a huge variety of fields, providing users with enriched and immersive visual learning experiences. In healthcare, AR and VR are utilized for training medical professionals and improving patient care through simulations and real-time data projections during procedures [25]. Virtual reality (VR) and mixed reality (MR) are effective educational tools in various healthcare settings. These tools are useful, such as preparing healthcare personnel to deal with disasters because they offer an immersive reality, as real-life exercises are very difficult to carry out in events with a large number of victims.

Virtual reality (VR) refers to a computer-generated, three-dimensional virtual environment that users can interact with, typically accessed via a computer that is capable of projecting 3D information via a display, which can be isolated screens or a wearable display, e.g., a HMD, along with user identification sensors. VR can mainly be divided into two categories: non-immersive, and immersive [54]. Non-immersive VR utilizes a combination of screens surrounding the user to present virtual information [20]. Immersive VR refers to using a wearable display, to track a user’s movement and present the VR information based on the position of users [48],which allows them to experience 360 degrees of the virtual environment.

There are certain factors of the VR system that could influence the user’s discomfort: Hardware factors such as display type, display mode, time delay, and content factors like variations in VR scenario by changing graphics or task-related features (e.g., duration and controllability) [8].

MR allows users to see the real world through head-mounted displays also, and overlays virtual elements [27]. Research indicates that immersive VR can lead to higher levels of mental and physical fatigue compared to non-immersive VR or real-world tasks. This phenomenon, often linked to intense sensory stimulation and cognitive overload, raises concerns about user well-being and performance [40].

VR immerses users in a fully digital environment, enhancing focus and concentration, making it particularly effective for simulations and scenarios that require deep cognitive engagement [13]. MR integrates digital elements into the real world, allowing users to interact with both physical and virtual objects, thereby promoting collaboration and real-time communication among learners. MR technology possesses profound and extensive potential across a multitude of domains, including healthcare, and education. However, prolonged use of MR devices to watch stereoscopic content may lead to visual fatigue [56].

The use of these technologies offers many benefits [2, 55],however, not all of the MR characteristics are advantageous. Thus, we observe important side effects of this type of innovative training, including so-called "virtual reality disease" [9, 17, 32, 47, 50]. Recent scientific work by Rolos and Merchant presents a systematic study of the different sickness vulnerability patterns observed in VR and MR HMDs [29]. They hypothesize that the reduction in motion sickness caused by MR is due to the translucent nature of MR HMDs, which allows partial visibility of the real world and facilitates visual-vestibular interaction that enhances congruence. About this syndrome, we focus on the symptom of fatigue, a concept that features different dimensions.

Fatigue has been widely studied in other situations [12, 43, 49]. One area of study related to this broad concept has been its relationship with the use of other new technologies, specifically concerning digital platforms [15, 38, 53]. However, given that our search did not reveal any articles that focused on measuring fatigue in the context of training in VR and MR, we designed a questionnaire to evaluate the level of fatigue perceived by professionals in this context. We validated this measure in the context of health emergencies after the use of VR and MR.

### Need for the research

Despite the exponential growth of immersive technologies in professional education—particularly in healthcare and emergency response—there has been limited exploration of the adverse cognitive and physical effects associated with VR and MR training [27, 50]. While numerous studies have validated the pedagogical advantages of these technologies in improving skill acquisition and decision-making under pressure [6, 33, 51], the potential fatigue effects they may induce have been largely overlooked. Fatigue, as a multidimensional construct encompassing physical, visual, emotional, motivational, and social domains, can significantly affect learning outcomes, engagement, and safety in training contexts [4].

In the specific context of mass casualty incident (MCI) training, immersive VR and MR environments provide highly realistic scenarios that are otherwise logistically and ethically difficult to reproduce in real life [3, 10]. However, the intense cognitive load and sensory stimulation inherent in such training can lead to fatigue symptoms that may hinder performance and reduce the benefits of prolonged exposure [19, 56]. Understanding and quantifying this fatigue effects are therefore essential to optimize training duration, prevent user discomfort, and ensure that immersive learning remains both effective and sustainable [9, 32].

The lack of a validated measurement instrument for fatigue within immersive simulation training highlighted a clear research gap. Existing tools, such as the Multidimensional Fatigue Inventory [49] and the Zoom Exhaustion and Fatigue Scale [15], were designed for different contexts. It has not been identified the specific sensory demand of disasters trainings with VR/MR.

This study was thus conceived to fill this methodological void by developing and validating the Virtual and Mixed Reality Fatigue Questionnaire (VMRFQ), a targeted instrument to assess professional fatigue profiles in immersive management disaster training. The study was conducted with HMDs of VR and MR.

The questionnaire developed constructs and dimensions for the scope of fatigue and assessed both previous experiences using VR, and the training time associated with this technology as factors that may affect the degree and intensity of fatigue in terms of its various dimensions.

Currently, there are several questionnaires with different perspectives related to fatigue items of VR/MR. The VMRFQ compared to these questionnaires used in VR/MR work offers this approach:

1. Simulator Sickness Questionnaire (SSQ) [44]: classic standard for simulator sickness/cyber sickness. It measures 16 items, three subscales (nausea, oculomotor, disorientation); developed for simulators and widely used in VR research. Strengths: long history, broad comparability between studies, well-understood scoring/weighting. Limitations compared to the VMRFQ: the SSQ focuses on nausea/disorientation and oculomotor symptoms (physiological motion sickness) rather than fatigue covering the mental/social/motivational area. The VMRFQ fills this gap by explicitly focusing on the motivational, emotional and social dimensions of fatigue that are not covered by the SSQ.
2. Virtual Reality Sickness Questionnaire (VRSQ) [28]: This is a tool similar to the VMRFQ: It assesses items adapted to VR symptoms (related to oculomotor movement). It is useful for studies of HMD exposure. The VRSQ focuses on the use of HMD and motion cues. Limitations compared to the VMRFQ: the VRSQ emphasises sensory/vestibular symptoms,it does not include the motivational items of social isolation, irritability or ‘too tired to do other things’ that are included in the VMRFQ.
3. Virtual Reality Neuroscience Questionnaire (VRNQ) [30] It covers measurements of users' virtual reality experience, including adverse effects (fatigue, among others), usability, and the quality of the software/hardware they use. This instrument seeks to unify the assessment of symptoms and usability in a single tool. Among its strengths: psychometrically developed with attention to modern HMD contexts. Among the limitations compared to the VMRFQ is the size of the questionnaire (combining usability, motion sickness, and immersion), while the VMRFQ is intentionally brief and focuses on fatigue dimensions relevant to training (an advantage for rapid follow-up after training).
4. Zoom Exhaustion & Fatigue (ZEF) Measures social/cognitive fatigue derived from video conferencing (loss of attention, social overload, self-perception, etc.). The ZEF explicitly includes social and motivational dimensions that conceptually overlap with the social and motivational elements of the VMRFQ. The inclusion of social fatigue in the VMRFQ is consistent with previous findings that mediated experiences produce social/exhaustion effects; it adapts that item specifically to immersive training in XR.
5. Adaptive fatigue detection model for virtual reality-based physiotherapy. It offers a physiological approach with a hybrid model. This model measures objectives correlates of fatigue (kinematic data from user interactions, identifying indicators of muscle fatigue). It proposes adaptive fatigue detection models for VR that combine behaviour and sensors. These are increasingly used for continuous monitoring in training in rehabilitation contexts. Martínez et al. [36] Among their strengths is that they provide objective validation, detecting the dynamics of fatigue during tasks within a rehabilitation environment with paraplegic patients.

## Method

**Fig. 1:**
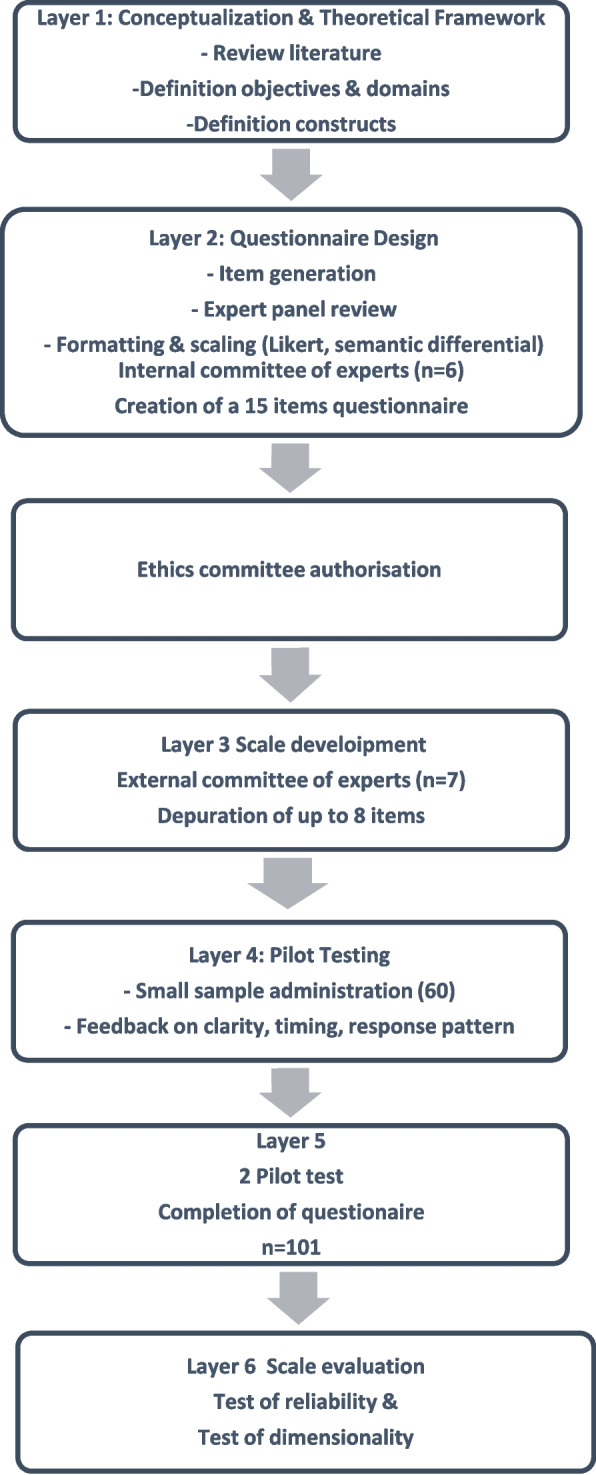
Diagram with layers of work — from conceptualization to final validation

The study consisted of a qualitative phase and a quantitative phase for the creation of the instrument and the collection of evidence for its validation, which lasted from 2022 to 2023 (Fig. 1).

Firstly, a review of the literature concerning this topic was performed.

The concept of fatigue is complex and multifaceted [14].The North American Nursing Diagnostic Association (NANDA) established a working definition of fatigue that identifies it as a feeling of exhaustion that is associated with a decreased capacity for physical and mental work [43, 54].

To account for the mental and physical aspects of fatigue, the Multidimensional Fatigue Inventory was examined [5]. This questionnaire represents one of the most widely used scales to measure fatigue and includes five constructs. Fauville [15] in the Zoom and Exhaustion Fatigue Scale (ZEF), defined five fatigue constructs as well. In the experts’ group, five constructs of fatigue that could be present in a MR experience were selected: General fatigue, Visual fatigue, emotional fatigue, social fatigue and motivational fatigue.

The first construct concern to general fatigue involves the manner in which people express their fatigue, is primarily through the phrase "I feel tired". Accordingly, general fatigue refers to the general experience of being tired. The second construct concerns how people express their visual fatigue as another construct related to fatigue. Social fatigue corresponds to a decreased level of activity that emerges frequently, associated with the time that people need to recover from the MR experience. The fourth dimension involves how people can experience fatigue in the form of difficulty concentrating. The construct of emotional fatigue involves the feeling of irritability.

These five dimensions of fatigue could contribute to our understanding of MR fatigue.

Moreover, engaging in VR or MR training alongside other professionals involves social interactions and speech. McCarthy & Saegert [37] proposed the concept of social overload based on the negative impact of crowded places that characterizes this type of training on the stress associated with properly addressing a Mass Casualty Incident (MCI) in a timely manner.

Training emergency teams to deal with a mass casualty event is challenging, as it involves recreating complex scenarios with multiple victims, medical staff with time-dependent manoeuvres such as triage and risks associated with the incident itself. The challenges of disaster management relate not only to the ability to handle uncertainty but also to the capacity to apply knowledge flexibly in dynamic and unpredictable conditions. Effective response requires adaptability in operations, decision-making, coordination processes, and the application of previously acquired competencies. Technical, non-technical, and internal factors—such as *to balance the mismatch between the disaster contingency plan and the reality, to establish a functional crisis organisation, to adapt the emergency medical response to an overall situation, to manage the staff and the stress*—can significantly influence medical management and decision-making, shaping how teams coordinate, communicate, and perform under pressure [22]. Response personnel may experience a range of emotions that can affect their performance [45].

Given the significant resources challenges associated with training large numbers of participants in disaster exercises, it is important to explore effective training methods for health professionals [4]. In this field, VR and MR technologies play a key role.

A descriptive and exploratory study focused on the validation of a fatigue questionnaire was conducted by experts by following the suggestions of Fehring [16] and employing the Delphi technique. This method used expert group validation as a basis for confirming the validity of the questionnaire.

The study had a national scope (Spain). Experts were defined as individuals who achieved a minimum score of six points based on the criteria proposed by Quatrini [42]. These criteria were related to clinical, teaching and research experience in critical healthcare. Additionally, the final inclusion criterion applied was knowledge of virtual/mixed reality and fatigue.

All the experts who showed interest in participating in the research were sent a letter of invitation, via e-mail, informing them about the project, detailing what their participation consisted of. Those who accepted the invitation were sent the link to the questionnaire, with the informed consent document. Anexx 1.

The selection of experts was made by non-probabilistic sampling; by convenience based on the network of contacts of the members of the research team and snowball sampling. The experts' years of experience range from 10 to 32 years, and the proportion was 2 men and 5 women.

The research team designed a questionnaire to be evaluated on-line by experts by Google Forms platform. We added several open-ended questions so that experts could comment on, remove, or add items. An adapted questionnaire from ZEF and Multidimensional Fatigue Inventory was sent out with 15 questions, of which 8 remained at the end of the evaluation and were used in this research (Appendix 1).

After various meetings were conducted and a consensus was reached among members of the research team and the external experts in fatigue (the council of experts, as described above), the final items to be included in the questionnaire were confirmed.

All surveys for experts and participants were created in the in the Google Forms© application, as was the survey itself to be validated. They were accessed via link sent by email which, together with information about the study's objective, contained an informed consent form that had to be ‘accepted’ in order to answer the questions in the surveys.

### Participants

One hundred and one prehospital emergency nurses, physicians and technicians from the Emergency Medical Service of Madrid (SUMMA112), who attended the professional Mass Casualty Incident (MCI) course. This course featured a VR segment lasting 15 min (the maximum time).

A nonprobabilistic convenience sampling approach was used for this research.

First, the participants received information regarding this research and signed an informed consent form.

Subsequently, they engaged in VR exercises with the assistance of Oculus Quest 2 goggles® and HoloLens® technology. These HMDs were used with physical mannequins in a second phase to display advanced training content using MR with other emergency professionals. Both VR HMDs provide an immersive modality. The wearable track the user’s movement and displays the VR information based on the position of users [48], This allows users to experience a full 360-degree view of the simulated setting.

The SUMMA112 professionals had previously participated in earlier versions of the Mass Casualty Incidents course as part of their ordinary corporate training. We freely invited participants in the VR practices to evaluate and complete the questionnaires after filling an inform consent.

We performed the initial VR procedures using two head-mounted displays (Oculus goggles® and HoloLens®) technologies indiscriminately. Both of these glasses feature a gyroscope, accelerometer and magnetometer [34, 39] and use stereoscopic content.

The data collection process took place between January 2023 and January 2024. The inclusion criteria limited the participants to emergency professionals (nurses, doctors, technicians or experts involved in health emergencies).

Our goal was to develop a tool that took into account both the psychological and physical aspects of fatigue related to VR and MR use.

To validate the fatigue questionnaire, a study featuring a quantitative approach and a nonexperimental design was conducted following the procedures associated with the adaptation of the ZEF scale and the development of the proposed Virtual and Mixed Reality Fatigue Questionnaire with the goal of obtaining evidence of the measure’s validity and reliability.

### Development of the research

In Table 1, we present the Virtual and Mixed Reality Fatigue Questionnaire.

**Table 1:** Fatigue Questionnaire for virtual and mixed reality

	Question	Answer
1	Do you feel fatigued after using virtual and/or mixed reality?	1- 2—3—4—5
2	Are you experiencing musculoskeletal discomfort such as neck pain or tension?	1- 2—3—4—5
3	Do you experience visual discomfort, such as pain, blurred vision, dryness, or irritation, after using virtual and/or mixed reality?	1- 2—3—4—5
4	Do you feel that you need time alone with yourself after using virtual..and/or mixed reality?	1- 2—3—4—5
5	Do you experience difficulty maintaining attention and concentration after using virtual and/or mixed reality?	1- 2—3—4—5
6	Do you feel too tired to do other things after using virtual and/or mixed reality?	1- 2—3—4—5
7	Do you feel emotionally drained after using virtual and/or mixed reality?	1- 2—3—4—5
8	How irritable do you feel after using virtual and/or mixed reality?	1- 2—3—4—5
9	How often do you practice virtual or mixed reality?	Never Approximately once per month Approximately once per week Approximately once per day Several times, more than once per day
10	How much time do you consider, in general, more suitable for training with mixed realityglasses?	1. 15 min 2. 20 min 3. 25 min 4. 30 min 5. 35 min
11	Do you consider the utilization of virtual reality to enhance the learning process to be necessary and/or useful?	1. Extremely necessary/useful 2. Very necessary/useful 3. Moderately necessary/useful 4. Slightly necessary/useful 5. Not necessary/useful at all

Each question from 1 to 8 was scored on the following five-point Likert scale:

1. Not at all.
2. Slightly
3. Moderately
4. A lot
5. Extremely

#### Procedure

The Virtual and Mixed Reality Fatigue Questionnaire was employed considering the ethical factors related to this research and ensuring that individuals could participate in this research in a voluntary, pseudoanonymous and confidential manner.

#### Data analysis

A confirmatory factor analysis (CFA) was performed using the maximum likelihood method. Finally, Cronbach's alpha coefficients were calculated to determine the reliability of the factors and the complete questionnaire.

To obtain validity evidence for the internal structure, confirmatory factor analysis (CFA) [26] is used, which allows us to verify the extent to which the data confirm the factorial model of the questionnaire generated from the theoretical foundation.

Thus, to test the reported model, a CFA was conducted, as this represents the ad hoc approach to studying the equivalence of versions of the same questionnaire in different languages [35]. Additionally, it allows us to contrast a previously constructed model, in which the researcher establishes a priori the full set of relationships between its constituent elements.

Several indicators were used to assess the model´ goodness-of-fit [21]: the chi- square statistic; the ratio of chi-square to the degrees of freedom (CMIN/DF); the goodness-of-fit index (GFI); the Tucker-Lewis index (TLI); the comparative fit index (CFI); and the root mean square error of approximation per degree of freedom (RMSEA) [7]. The chi-square statistic indicates the absolute fit of the model but is very sensitive to sample size. Therefore, the ratio of chi-square to the degrees of freedom is usually also interpreted, with values ​​below 3 indicating a good fit. The GFI, TLI, and CFI indices range from 0 to 1, with 0 indicating no fit and 1 indicating optimal fit. Values ​​of 0.95 or higher are considered excellent, and values ​​above 0.90 suggest an acceptable fit of the model to the data [46]. The RMSEA index is considered optimal when its values ​​are 0.05 or lower, and acceptable in the range 0.05–0.10 [7].

The data were analyzed using SPSS V.28.0 software.

## Results

Cronbach's alpha was calculated for the collected data. This is an indicator of adequate reliability assessment, as the construct was tested unidimensionally. This analysis yielded acceptably high reliability levels, with α = 0.878 for the construct's eight items. As seen in Table 2, this coefficient does not increase when any item is removed from the Questionnaire.

**Table 2:** presents the eight items across the five constructs that form the final questionnaire

Constructs	Question
General Fatigue	Do you feel fatigued after using virtual and/or mixed reality?
	Are you experiencing musculoskeletal discomfort such as neck pain or tension?
Visual Fatigue	Do you experience visual discomfort, such as pain, blurred vision, dryness, or irritation, after using virtual and/or mixed reality?
Social Fatigue	Do you feel that you need time alone with yourself after using virtual and/or mixed reality?
Motivational Fatigue	Do you experience difficulty maintaining attention and concentration after using virtual and/or mixed reality?
	Do you feel too tired to do other things after using virtual and/or mixed reality?
Emotional Fatigue	Do you feel emotionally drained after using virtual and/or mixed reality?
	How irritable do you feel after using virtual and/or mixed reality?

It is noteworthy that the item-total correlation is, in all cases, high (*r ≥* 0.45) and statistically significant (*p <* 0.001), maintaining the internal consistency characteristics of the original version (Table 2).

To discuss fit, we must consider the criteria for the various model indices. The model demonstrated reasonable fit according to the chi-square test (χ^2^ = 61.10, gl = 69, *p <* 0.001) and the CMIN/DF = 1.08 (< 3). The value obtained for the root mean square error of approximation (RMSEA = 0.05) is below the reasonable limit (0.10) [7]. However, the values ​​of GFI = 0.65, CFI = 0.71 and TLI = 0.639 are below 0.9 for a good fit6, but it is known that CFI and TLI depend on the sample size. Since there is no agreement on the values ​​that a good fit should provide in all indices or measures,

### Findings- psychometric analysis

Table 3 represents all the statistics used with the results of the estimation of these parameters together with their recommended values, which are more widespread in the literature for the assessment of the model fit [7, 35]. Based on these indexes, this sample has an acceptable fit to the two-factor model.

**Table 3:** Total-item statistics results

	**Average** of the questionnaire if the item is removed	**Variance** of the questionnaire if the item is removed	**Corrected item**-to-total correlation	**Cronbach's alpha** if the item is removed
Question 1	10.27	12.158	0.483	0.886
Question 2	10.81	12.954	0.527	0.874
Question 3	10.48	11.572	0.613	0.869
Question 4	10.80	12.180	0.658	0.861
Question 5	10.76	12.463	0.715	0.857
Question 6	10.82	11.768	0.808	0.846
Question 7	10.85	12.288	0.727	0.855
Question 8	10.87	12.473	0.736	0.856

Table 4 shows the indexes results.

**Table 4:** Summary of the model's coefficients and goodness-of-fit indexes

Statistics		Recommended adjustment level	Adjustment level obtained
Chi-square test (χ^2^)		p > 0.05	61.0; df = 69; *p <* 0.001
Fit summary model (Absolute fit)	CMIN/DF	Approx. 2–5	1.08
Baseline Comparisons (comparative fit)	GFI TLI CFI	> 0.9	0.65 0.639 0.71
Others	RMSEA	< 0.05 (good fit) 0.05–0.08 (acceptable fit)	0.05

CMIN/DF: χ^2^/df NFI: Normed Fix Index, *GFI* Goodness-of-fit index, *TLI* Tucker-Lewis Index, *CFI* Comparative Fit Index, *RMSEA* Root Mean Square Error of Approximation

Table 5 shows the average, median and standard deviation of each Fatigue item, obtained by analyzing the questions results of the questionnaire.

**Table 5:** Statistics measures of central tendency

	Descriptive statistics			
Number of question	**Average**	**Median**	**Standard deviation**	**Sample size**
Question 1	1.97	2	0.842	101
Question 2	1.43	1	0.622	101
Question 3	1.76	2	0.826	101
Question 4	1.44	1	0.670	101
Question 5	1.48	1	0.576	101
Question 6	1.42	1	0.637	101
Question 7	1.39	1	0.6	101
Question 8	1.37	1	0.561	101

An analysis of the data revealed that after training in MCI using VR/MR technology, The general fatigue got the maximum average with 1.97. The median of 2 aligned with the standard deviation marked in general fatigue and visual discomfort the key points.

Table 6 shows the results regarding participants’ previous experience in virtual or MR, 87.1% of the volunteers had never used VR or MR, 4% reported using it once per week, and 3% reported using it daily.

**Table 6:** Descriptive size for the MR/VR trainees experience, the monullre adequate training time according to user experience and the perceived usability of the MR/VR professional training

How often do you practice virtual or mixed reality?		How much time do you consider, in general, more suitable for training with mixed reality glasses?		Do you think it is important/useful to use mixed/virtual reality to improve learning in Mass Casualty Incident training?	
Never	88	15 min	33	Extremely important	36
Approximately once a month	6	20 min	51	Very important	34
Approximately once a week	4	25 min	4	Moderately important	31
Approximately once a day	1	30 min	13	Slightly important	0
Several times a day	2	35 min	0	Not important at all	0

A total of 50.5% of the participants identified the length of VR training ranging to 20 min as adequate. A minority of 12.8% of the participants would prefer 30 min of training time (question 10).

When we asked the participants about the usefulness of this technology in terms of its ability to enhance the learning process, 31 participants considered it to be moderately important, 34 considered it to be very important, and 36 considered it to be extremely important; no participants evaluated this pedagogical tool as exhibiting little value.

## Discussion

The use of a methodology based on VR/MR in attempts to instruct health staff in how to respond to incidents involving multiple victims is acceptable in terms of the fatigue it produces. This approach can be an interesting teaching tool if the proper training time to obtain optimal results is taken into account and if secondary effects are avoided as much as possible.

We have found systematic reviews that analyses the effectiveness of this type of training of medical first responders for preparing to resolve MCI [3, 10]. New lines of research comparing VR/MR-induced fatigue with other training methods are suggested, as well as the applicability of the questionnaire in different contexts.

This approach represents an improvement over current and traditional training methods and is more effective in the long term due to its increased capacity to generate immersive simulations that can offer trainees in MCI courses the most realistic experience of the task of performing in the context of an MCI.

A significant proportion of participants had not previously used XR. Although this limits the data from direct experience, it offers insight into how new users might respond to XR technologies, an important consideration for educators introducing these tools into their practice. The specific types of fatigue that were most prominently identified were the general fatigue, visual fatigue and the motivational fatigue. The participants who frequently used VR suffered less fatigue than people who were experiencing VR for the first time, especially for eye-related symptoms, emotional symptoms and irritation. Physical discomfort and other negative factors can most likely be mitigated if healthcare professionals are accustomed over time to the use of VR/MR. In most cases, these data were obtained from healthcare professionals who are not accustomed to VR. This hypothesis could be confirmed by additional studies that can corroborate this trend.

The questionnaire has demonstrated consistency and strength in the different constructs, with all items showing a strong linear correlation. This reflects the nature of the tool itself, beyond the expected fatigue results from the use of XR, where visual and musculoskeletal effects were anticipated, but not so much emotional, motivational, or social effects.

We have found systematic reviews that analyse the effectiveness of this type of training. Del Carmen Cardós-Alonso et al. [10, 41] New lines of research comparing VR/MR-induced fatigue with other training methods are suggested.

Another factor that should be considered in the future is fatigue affectation, particularly about whether fatigue levels change based on the purpose of MR use, such as whether this approach is used for training at the professional level, as in the case, or for entertainment-related purposes. Measurements in these different environments may vary due to a variety of factors, such as the responsibility associated with learning in the professional environment or the increased challenge of interpreting visual data in an immersive environment. It might also be useful to compare the benefits and adverse effects of immersive and non-immersive forms of MR in this type of training.

The type of headset display used in this context can influence the results of such a fatigue evaluation based on the immersive experience of the field trial and its quality. Although a research has found MR HDM to be less sickness [29], it still causes fatigue. More researchers are needed to confirm this trend following the improvement of new MR HDM models in the future.

The integration of VR and MR into healthcare settings has sparked ongoing debates among managers, researchers, and technology developers regarding their effectiveness and implications. Concerns about diminishing face-to-face interactions, the psychological effects of immersive technologies, and the adequacy of training for educators remain prominent points of contention [11, 24, 57]. As health institutions continue to explore these technologies, understanding their differences and potential drawbacks is vital for leveraging their benefits while mitigating adverse effects.

Ultimately, both VR and MR represent transformative possibilities in professional education, but their successful implementation requires careful consideration of their unique characteristics, challenges, and the diverse needs of learners. Future research is essential to optimize these technologies for professional improvement purposes, ensuring they are effectively utilized to enhance learning experiences while minimizing potential negative impacts on trainees’s cognitive and emotional well-being [31, 51].

The training of healthcare and emergency personnel using virtual and mixed reality (VR/MR) technologies has gained importance due to its ability to safely and repeatedly simulate complex and high-risk situations. However, despite the growing integration of immersive systems into education and disaster preparedness, little attention has been paid to the human impact of these technologies, particularly the onset of fatigue associated with prolonged use of VR/MR. Existing fatigue assessments, such as the MFI and the ZEF, were not designed to capture the unique sensory, cognitive, and emotional load imposed by immersive environments in training emergency professionals to manage mass casualty incidents. Consequently, there was a clear need to develop a specialised instrument capable of assessing fatigue across multiple dimensions, specifically in the context of emergency professionalisation using virtual and mixed reality.

### Novelty of the work

The present study introduces the Virtual and Mixed Reality Fatigue Questionnaire (VMRFQ), a novel and validated tool designed explicitly to measure fatigue derived from immersive training environments in health emergencies in MCI. Unlike previous instruments, the VMRFQ integrates the physical, visual, motivational, emotional, and social dimensions of fatigue, reflecting the complex interaction between sensory distress, attentional demands, and psychological factors characteristic of VR/MR experiences in professional emergency training environments. Furthermore, this work advances methodological rigour in this field by combining exploratory and confirmatory factor analyses (EFA and CFA) to establish construct validity and internal consistency.

### Features where VMRFQ is innovative/advantageous

- *Focus on fatigue (not just cybersickness)*: explicitly captures motivational, emotional and social fatigue — dimensions under-represented in SSQ/VRSQ. This is valuable for professional training outcomes (attention, emotional drain, readiness to perform).
- *Brevity and training orientation*: 8 core items + a few practical items (frequency of XR exposure, preferred duration, perceived usefulness) — fast to deploy immediately after short training modules. Good for operational use in courses.
- *Direct applicability to MCI/emergency training*: validated in the target population (prehospital emergency staff), which strengthens ecological validity for that domain.
- Short comparison table between VMRFQ and other main questionnaires related to the study topic.

**Table:** 

Aspect	VMRFQ	SSQ/VRSQ/VRNQ
Focus	Multidimensional fatigue (general, visual, social, motivational, emotional)	Cybersickness (nausea, oculomotor, disorientation) and usability
Length	Very short (8 items) + usability/time items	SSQ: 16 items; VRSQ/VRNQ: variable (short → medium)
Best use	Quick post-training monitoring in courses (esp. MCI/emergency)	In-depth sickness profiling and cross-study comparisons
Psychometrics (so far)	α = 0.878; CFA RMSEA ≈ 0.05; CFI/TLI low; N = 101 (mostly with little experience in XR)	Well-established norms for SSQ; VRNQ report robust psychometric evidence in larger samples
Gaps	Needs larger samples, test–retest	Gaps: SSQ less sensitive to social/motivational fatigue

### Key limitations and future research directions

As limitations, we found limitations in the sample and generalisation: convenience sampling (n = 101) and the high percentage of people with no previous experience with extended reality (~ 87%) limit generalisation. It would be advisable to extend the study to larger cohorts, with mixed experiences and professional populations from other emergency groups (firefighters, police officers, etc.). Another future synergistic study could cover a comparation with a group using flat-screen VR. Other lines of research are proposed in which the diversity and size of the sample are increased, with validation at multiple sites with participants with mixed experience in XR (novices + experienced), including non-emergencies-related professionals and control groups (flat screen training) to test the specificity of immersion.

Given the previous experience of the study population, it is worth testing whether the questionnaire is stable over time (when there is no new exposure) and sensitive to variations in exposure duration/intensity (15 vs. 30 min, immersive vs. non-immersive).

### Main conclusions

The study demonstrated that the VMRFQ has robust psychometric properties, with high internal reliability (α = 0.878) and satisfactory model fit indices (e.g., RMSEA < 0.08, CFI and TLI > 0.90). The five-factor structure (general fatigue, visual fatigue, social fatigue, motivational fatigue, and emotional fatigue) was empirically supported, indicating that fatigue in immersive contexts is multifaceted. The results revealed that prolonged exposure to VR/MR can cause measurable fatigue effects that influence concentration, motivation, and emotional regulation, which in turn can affect performance and learning retention.

The results underscore the importance of monitoring user well-being and optimising session duration, interface design, and ergonomic conditions in extended reality-based training. Beyond its immediate application in healthcare and emergency simulations, the VMRFQ lays the foundation for future interdisciplinary studies exploring human adaptation and resilience in professional immersive environments.

The main value of this questionnaire and its use in this research lies in the fact that, unlike existing literature, which focuses solely on visual fatigue, we analyse the different dimensions of fatigue that affect the optimal use of interactive mixed reality learning for healthcare professionals. Motivational, emotional, and social fatigue are in this order the most statistically significant.

This validated scale (Virtual and Mixed Reality Fatigue Questionnaire) is useful for detecting the general, social, emotional, visual, and motivational dimensions of fatigue. The fatigue questionnaire for VR and MR fulfills several objectives; on the one hand, it provides a method for subjectively measuring experienced fatigue in the context of this training model and makes it possible to optimize various aspects such as the duration of training or the experience of well-being in the context of MR, thus allowing us to make this approach more effective.

The duration of the VR/MR activity was also identified as a decisive factor that influenced the results obtained by applying the fatigue questionnaire.

Despite the emergence of mild symptoms, users identified a duration of 15–20 min as optimal for an immersive VR experience.

In conclusion, it is crucial to establish a balance among immersion, influencing factors and fatigue to optimize participants’ experience in these environments. Given the increase in the use of new technologies, such as VR&MR, this work is focused on determining the validity of a questionnaire used to measure fatigue when these technologies are used.

After controlling certain variables, data were collected to adapt this questionnaire and observe participants’ responses to VR/MR training fatigue. The VR and MR Fatigue Questionnaire includes validated constructs about physical and mental fatigue. The test is an adequate measurement of the fatigue items that were proposed.

The increase in the use of this technology requires these measurements to perform the best professional training using VR or MR.

## Supplementary Information

Supplementary Material 1

## Data Availability

The data and materials are available at [https://bit.ly/DatabaseSUMMA112ValidationFatigueResearch] (https:/bit.ly/DatabaseSUMMA112ValidationFatigueResearch).

## Appendix 1

**Table 7:** Constructs of the VMRFS and their associated Items

Construct / Variable	Definition / Description	Associated Question(s)
GF	General Fatigue: Overall feeling of physical or mental tiredness resulting from prolonged use of virtual and/or mixed reality environments.	• Do you feel fatigued after using virtual and/or mixed reality?• Are you experiencing musculoskeletal discomfort such as neck pain or tension?
VF	Visual Fatigue: Eye strain or visual discomfort caused by prolonged exposure to immersive displays (HMDs), including symptoms such as pain, blurred vision, dryness, or eye irritation.	• Do you experience visual discomfort, such as pain, blurred vision, dryness, or irritation, after using virtual and/or mixed reality?
SF	Social Fatigue: The perceived need for solitude or reduced social interaction following virtual or mixed reality experiences.	• Do you feel that you need time alone with yourself after using virtual and/or mixed reality?
MF	Motivational Fatigue: Decrease in motivation, interest, or ability to maintain focus following exposure to virtual and/or mixed reality.	• Do you experience difficulty maintaining attention and concentration after using virtual and/or mixed reality?• Do you feel too tired to do other things after using virtual and/or mixed reality?
EF	Emotional Fatigue: Emotional exhaustion or irritability experienced after using immersive technologies.	• Do you feel emotionally drained after using virtual and/or mixed reality?• How irritable do you feel after using virtual and/or mixed reality?

**Table 8:** Nomenclature of abbreviations and variables used in the study

Abbreviation/Symbol	Definition/Description
AR	Augmented Reality – integration of virtual elements into the real-world environment.
CFA	Confirmatory Factor Analysis – statistical technique used to validate the internal structure of a scale.
CFI	Comparative Fit Index – compares model fit with a null (independence) model.
CMIN/DF	Chi-square to degrees-of-freedom ratio; values < 3 indicate good model fit.
EFA	Exploratory Factor Analysis – used to identify underlying relationships between measured variables.
FIIBAP	Fundación de Investigación e Innovación Biosanitaria de Atención Primaria.
GFI	Goodness-of-Fit Index – measures the relative amount of variance and covariance explained by a statistical model.
HMD	Head-Mounted Display – wearable visualization device enabling immersive XR experiences.
MCI	Mass Casualty Incident – emergency event involving multiple victims requiring coordinated health response and triage.
Med1stMR	Medical First Responder Training using a Mixed Reality Approach featuring haptic feedback for enhanced realism.
MFI	Multidimensional Fatigue Inventory – standardized measure of fatigue across multiple dimensions.
MR	Mixed Reality – merging of physical and virtual environments allowing real-time interaction.
N	Sample size – number of participants included in the analysis.
NANDA	North American Nursing Diagnosis Association – defines the nursing diagnosis of fatigue used in conceptual framework.
NFI	Normed Fit Index – assesses improvement in fit compared to a null model.
p	Probability value used to test statistical significance; p < 0.05 indicates significance.
RMSEA	Root Mean Square Error of Approximation – assesses model fit per degree of freedom; values < 0.08 indicate acceptable fit.
SD	Standard Deviation – measure of data dispersion around the mean.
SPSS	Statistical Package for the Social Sciences – software used for data processing and statistical analysis.
SUMMA112	Servicio de Urgencias Médicas de Madrid SUMMA112 / Emergency Medical Services of Madrid Community.
TLI	Tucker-Lewis Index – evaluates model fit adjusted for model complexity.
VMRFQ	Virtual and Mixed Reality Fatigue Questionnaire – instrument developed to measure perceived fatigue during VR/MR professional training.
VR	Virtual Reality – immersive computer-generated environment that replaces the real world.
VRNQ	Virtual Reality Neuroscience Questionnaire
VRSQ	Virtual Reality Sickness Questionnaire
XR	Extended Reality – umbrella term including Virtual Reality (VR), Augmented Reality (AR), and Mixed Reality (MR).
ZEF	Zoom Exhaustion & Fatigue Scale – validated tool assessing fatigue in virtual meetings.
α (Cronbach’s Alpha)	Reliability coefficient measuring internal consistency of a scale; higher values indicate stronger reliability.
