# Supplementary material for: Virtual & mixed reality fatigue questionnaire
Source: BMC Med Educ. 2026 Feb 1;26:373. doi: 10.1186/s12909-026-08641-w (PMC12958750; doi:10.1186/s12909-026-08641-w)
Supplement: Supplementary file 1 — Supplementary Material 1 [file 12909_2026_8641_MOESM1_ESM.docx]

ANEXX 1 Characteristics and contributions of the expert group

| **EXPERT & LEVEL OF EDUCATION** | **WORKPLACE** | **YEARS OF EXPERIENCE** | **GENERAL COMMENTS** | **MODIFICATION OF QUESTIONS** | **QUESTIONS REMOVED** | **QUESTIONS ADDED** |
| --- | --- | --- | --- | --- | --- | --- |
| 1 RN, Podiatrist, Anthropologist, PhD, several MSc.  Expert in the use of VR/MR. | University Professor and emergency nurse | 22 years as a nurse and 16 years as a university professor |  | “Question 10. I don't see it very clear (“you fear having to do things”)” | “Suggestion: I see questions 5 and 6 as too similar. I think it is more common for question 6 to occur, so I would eliminate question 5.  Questions 8 and 9 are too similar. I would eliminate question 9.  Questions 11 and 12 are too similar. Eliminate one.” | “Suggestion:  Do you feel dizzy or lightheaded after VR/MR?  Do you have difficulty maintaining attention and concentration after the VR/MR session?  do you feel sleepy/want to sleep after VR/MR?” |
| 2 Doctor, Phd  Expert in VR and MR. | University Professor | 32 | Several questions are quite similar and can be unify | Unclear questions: 1,2,3,9,13 and 14. In the “unclear” items, define the concept evaluated. | Question 15: How grumpy do you feel after using virtual and/or mixed reality? |  |
| 3 PhD in Psychology  Expert in VR and MR. | University Professor | 10 | “Suggestion:  In general I find no problems related to the order.” | “According to the context of the instructions, it is indicated that it is for the use of Zoom, but would it work for another videoconferencing system as well? If this is the case, the instructions given to the participants need to be reviewed.  Also, I notice that virtual/mixed reality is mentioned all the time, but Zoom is a videoconferencing system, it is necessary to clarify what is meant by virtual reality.  It is also convenient to check if the instrument intends to evaluate the last videoconference or something in general. Because if this is the case, there could be two measurements, the situation and something more stable.  The format of the ordinal responses may give problems to some users, perhaps you can leave the first and last category with name and then only leave numerical values.” | “Suggestion: There are items with very similar content, it is better to eliminate them to avoid that the internal consistency is falsely increased by the inclusion of similar items and not by a correct mapping of the construct. The gradation of the responses of these similar items is already implicit in the item format (graded/ordinal response).  Item 7 does not seem to be related to what it is intended to measure.” | “Suggestion: it is suggested to group the questions by dimensions to check if, indeed, there are enough items that capture well the definition of each dimension.  I miss some item on musculoskeletal discomfort such as neck pain, tension, etc.” |
| 4 PhD in Psychology, specialist in Positive Psychology and Sense of Humor. Master in Psychology of Physical Activity and Sport. Expert in fatigue | University Professor | 17 years as a sports psychologist and 14 years as a university lecturer. | “Suggestion:  The order is fine.” Some questions perhaps are not needed. | “Suggestion:  Do you feel dizzy or lightheaded after VR/MR?  Do you have difficulty maintaining attention and concentration after the VR/MR session?  Do you feel sleepy/like you want to sleep after VR/MR?”    These you have raised I like, but it would be at best adding a new dimension to the original scale.” | Original item  8 How much do you want to be alone after the use of virtual and/or mixed reality?  9 How much do you need for yourself after the use of virtual and/or mixed reality?  10 How much do you dread having to do things after virtual and/or mixed reality use? | “Suggestion:  8. Do you like to be alone after the use of virtual and/or mixed reality?  9.Do you need time for yourself after virtual and/or mixed reality use?  10. “Do you feel like doing things after virtual and/or mixed reality use?  Although it would be reversed and doesn't take into account anticipation.” |
| 5 PhD in Neuroscience. | Foundation for Biosanitary Research and Innovation in Primary Care.  University Professor | 20 years as psychology professional and university lecturer |  | The number of items can be reduced to avoid redundance, from 15 items to 8; maintenance the similar constructs. | Some constructs can present 2 items and others only one that is enough. |  |
| 6 Psychology, Master in Psychology of Physical Activity and Sport. | University Professor |  |  | The response scale is still not uniform for all items (the motivational fatigue items use a different response scale than all other items) and we found that the two items you chose for each scale are partly not selective. For example, the two general fatigue items relating to tiredness and burnout show very similar constructs. We suggest that you re-examine all items belonging to each subscale and choose the two most selective items for each construct. | The questions 2,4,6,8,12,13,14 can be eliminated, the constructs are represented by the others questions. |  |
